# Novel photonic crystal fibre for low-noise coherent supercontinuum generation

**DOI:** 10.1038/s41598-026-43460-5

**Published:** 2026-03-25

**Authors:** R. Morel, J. Millo, N. Forget, V. Thibaut, M. Marcadier, A. Jullien, Y. Pertot, A. Cassez, V. Andrieux, D. Labat, O. Vanvincq, A. Kudlinski, J. M. Dudley, T. Sylvestre

**Affiliations:** 1 Université Marie et Louis Pasteur , SUPMICROTECH, CNRS, Institut FEMTO-ST, Besançon, France; 2 Université Côte d’Azur, CNRS, Institut de Physique de Nice (INPHYNI), Nice, France; 3 Fastlite by Amplitude Systems, Antibes, France; 4 Université de Lille, CNRS, PhLAM-Physique des Lasers Atomes et Molécules, Lille, France

**Keywords:** Photonic crystal fibres, Nonlinear optics, Fibre optics, Supercontinuum generation, Optics and photonics, Physics

## Abstract

We present a polarisation-maintaining all-normal dispersion photonic crystal fibre designed for 1030 nm femtosecond pumping, enabling ultra-stable and coherent supercontinuum (SC) generation spanning 650–1300 nm. The fibre’s polarisation-maintaining properties are achieved through two larger central holes in the structure, which is an alternative approach to using conventional stress rods. The fibre is specifically engineered to achieve minimum dispersion near 1030 nm, making it ideal for ultrafast comb-based metrology, and widely tunable optical parametric amplifier (OPA) systems. We further investigate the influence of input pulse contrast on supercontinuum generation through both numerical simulations and experiments. Relative intensity noise (RIN) and phase noise (PN) are characterized using three complementary techniques: dispersive Fourier transform (DFT), the Bellini–Hänsch interferometric method, and the dual-reference oscillator cross-correlation technique. The results demonstrate excellent stability, with pulse-to-pulse RIN below 0.5%, an optical phase deviation under 15 mrad, and phase noise levels down to − 150 dBc/Hz at 10 kHz from the carrier, confirming the fibre’s suitability for demanding ultrafast applications.

## Introduction

Supercontinuum generation (SCG) in all-normal dispersion (ANDi) optical fibres has attracted growing interest in recent years, owing to its capacity to deliver broad, flat spectra with high brightness, excellent stability, and high coherence^1–6^. These low-noise SC sources are critical for a wide range of applications, including optical coherence tomography (OCT), coherent combining, nonlinear imaging, ultrafast optics, and dual-comb spectroscopy operating at the shot-noise limit^7–9^. Unlike soliton-based SCG, which suffers from coherence degradation due to noise-seeded modulation instability, soliton collisions, and stimulated Raman scattering (SRS), ANDi SC generation develops from nonlinear dynamics governed by self-phase modulation (SPM) and optical wave breaking (OWB), resulting in highly coherent and low-noise spectral broadening^1–4^. ANDi-fibre based SC sources have already demonstrated significant impact, including the realization of the first shot-noise-limited OCT and GHz-rate dual-comb systems^7^. Furthermore, combining soliton self-compression with OWB has enabled octave-spanning SC spectra with exceptional noise properties, even under low-energy pumping^10^. The broadband, compressible chirped pulses generated in ANDi fibres are also well suited for generating low-noise, single-cycle pulses, important for applications such as high-field and attosecond science^11^.

Despite these advantages, ANDi-Fibre-based SCG is often constrained by polarisation instabilities such as polarisation modulation instability (PMI) and SRS, which limit the usable fibre length or pulse duration^12^. To mitigate these effects, polarisation-maintaining (PM) photonic crystal fibres (PCFs) have been developed, offering improved polarisation stability and high polarisation extinction ratios (PERs)^13^. However, conventional silica-based PM-ANDi fibres typically rely on stress rods and require a high number of air-hole rings with low air-filling fractions, making fabrication complex.

In this paper, we introduce a simplified PM-ANDi silica-based PCF design that achieves polarisation maintenance via two enlarged central air holes, offering an alternative to conventional stress-rod designs. The fibre is specifically engineered to achieve minimum dispersion near 1030 nm, making it ideal for SC generation from Ytterbium femtosecond lasers. Using this novel fibre design, we demonstrate flat and symmetric SC spectra spanning from 630 to 1350 nm, generated by two different femtosecond laser sources. We further evaluate the stability of the SC source through pulse-resolved relative intensity noise (RIN) measurements using the dispersive Fourier transform (DFT) and phase noise characterization in both the optical and electrical domains, confirming its excellent shot-to-shot stability.

### All-normal dispersion photonic crystal fibre

**Fig. 1:**
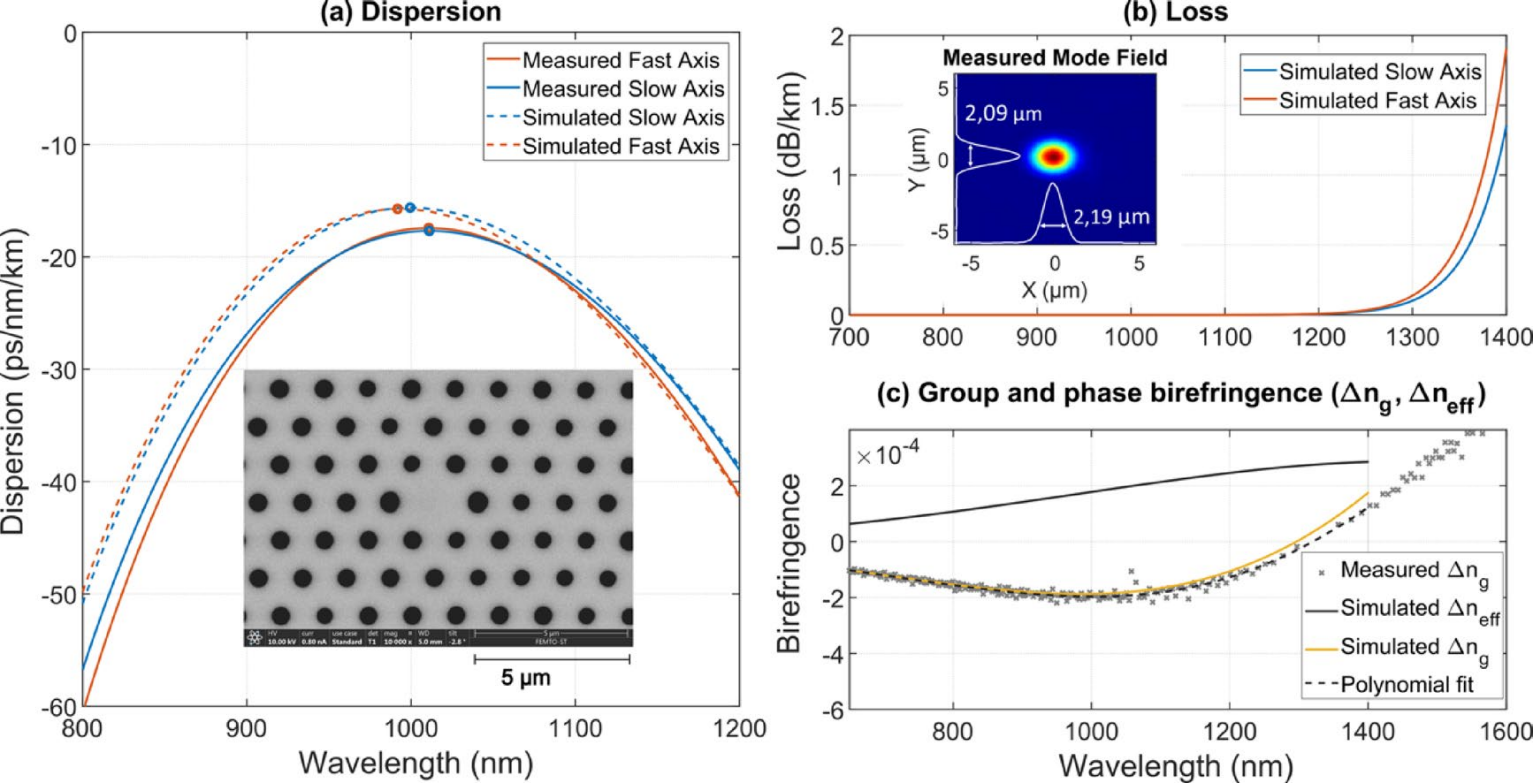
Characterization of the polarisation-maintaining all-normal-dispersion (PM-ANDi) photonic crystal fibre (PCF) designed for low-noise coherent SC generation. (**a**) Measured and simulated group-velocity dispersion (D) for both principal axes. The inset presents a scanning electron microscope (SEM) image of the fibre microstructure. (**b**) Simulated confinement losses for the two principal polarisation axes. The inset shows the measured mode-field intensity distribution. (**c**) Comparison between measured and simulated group (Δn_g_) and phase (Δn_eff_) birefringence.

Figure 1a shows a scanning electron microscope (SEM) image of the photonic crystal fibre (PCF) cross section. The fibre was manufactured using the standard stack and draw method by stacking more than 400 silica glass capillaries. It features 11 rings of air holes in order to limit confinement losses to a few dB/km for wavelengths shorter than 1300 nm, as shown in Fig. 1b. The fibre core has a diameter of 2.3 µm, and the air-filling fraction (d/Λ) is measured to be 0.39, ensuring single-mode operation across the whole supercontinuum bandwidth, as shows the measured output mode-intensity profile in inset of Fig. 1b. By precisely optimizing the fibre pitch (Λ = 1.42 µm) and the diameter of the smaller holes (d₁ = 0.55 µm), a smooth, parabolic ANDi profile was achieved, with the minimum dispersion wavelength (MDW) centered at 1010 nm (See Fig. 1a). Figure 1a shows the measured and simulated dispersion profiles for both the slow (blue) and fast (red) axes of the PM fibre. The simulations were performed using a finite element method (FEM) solver, based on the real SEM image to set the hole radii and pitches. Dispersion measurements were then carried out with a custom Mach–Zehnder interferometer and a broadband supercontinuum source (LEUKOS Electro-Vis). The experimental procedure for dispersion measurements follows a methodology similar to that described in Ref.^14^.

The polarisation-preserving characteristics are realized through two larger holes (d₂ = 0.64 µm) positioned symmetrically adjacent to the core (See inset of Fig. 1a), leading to a birefringence as high as 2 × 10^−4^ at 1030 nm (See Fig. 1c). This PCF design does not require the use of stress rods to obtain high birefringence so that the fibre outer diameter can be reduced to 120 µm, as compared 200 µm for commercially-available ANDi fibres using stress rods (NKT NL-PM-1050-NEG^15^). This allows easier cleaving and connectorization. The phase birefringence shown in Fig. 1c was obtained from numerical simulations based on the measured SEM image of the fiber cross-section. In contrast, the group birefringence was measured experimentally using a broadband polarimetric method. The setup consisted of a supercontinuum laser source (LEUKOS Electro-Vis) combined with two broadband polarizers (LPVIS050, Thorlabs). The interference between the two orthogonal polarization modes was analyzed spectrally, allowing us to retrieve the wavelength-dependent group birefringence from the spectral modulation period.

### Supercontinuum numerical modeling

To simulate fs-pumping SC generation within the PM-ANDi fibre, we used the scalar generalized nonlinear Schrödinger equation (GNLSE) in the following form^2–6^:

1 $$\frac{\partial A}{{\partial z}} + \frac{\alpha (\omega )}{2}A - \sum\limits_{n \ge 2} {\frac{{i^{n + 1} }}{n!}} \beta_{n} \frac{{\partial^{n} A(z,T)}}{{\partial T^{n} }} = i\gamma \left( {1 + i\tau_{0} \frac{\partial }{\partial T}} \right)\left( {A\int_{ - \infty }^{\infty } R (T^{\prime})|A(z,T - T^{\prime})|^{2} dT^{\prime}} \right),$$

where *A(z, T)* is the complex amplitude of the pulse propagating in the *z* direction, *T* is comoving time (in a reference frame moving at the group velocity at 1030-nm) $\alpha (\omega )$ is the frequency-dependent confinement loss, and $\beta_{n}$ is the *nth* derivative of the propagation constant. The nonlinear coefficient $\gamma = {2}\pi n_{{2}} /(\lambda_{{\mathrm{P}}} A_{eff} )$ is obtained using $n_{{2}}$ = 2.18 ×10^−20^ *m*^2^/*W* for the nonlinear refractive index of the silica glass at 1030 nm^16^. The effective area $A_{eff}$ of the fundamental mode was estimated from FEM simulations, yielding $\gamma = {3}0W^{{ - {1}}} km^{{ - {1}}}$ (at 1030 nm), a value approximately 10 times larger than that of a standard (SMF28) silica fibre. The temporal derivative of the field envelope on the right side of in Eq. (1) corresponds to the self-steepening effect with its characteristic time scale $\tau_{0} = { 1}/\omega_{0}$ (ω_0_ being the angular frequency at 1030 nm). $R(T) = ({1} - f_{R} )\delta(T) \, + f_{R} h_{R} (T)$ is the nonlinear response, where $h_{R} (T)$ is the delayed temporal Raman response^17^, and $f_{R}$ the fractional contribution of the Raman effect. We use $f_{R} = 0.{18}$ corresponding to standard silica fibre^17^. We also add quantum noise in our numerical simulations using one photon per mode with a random phase^18^. In addition, propagation and confinement losses, both of which become significant beyond 1300 nm, are taken into account. It should also be noted that we employ the scalar GNLSE in this work, as we model propagation only along each principal axis of the fibre.

### Experimental and numerical results

#### Pumping at 1034 nm with 175 fs pulses

**Fig. 2:**
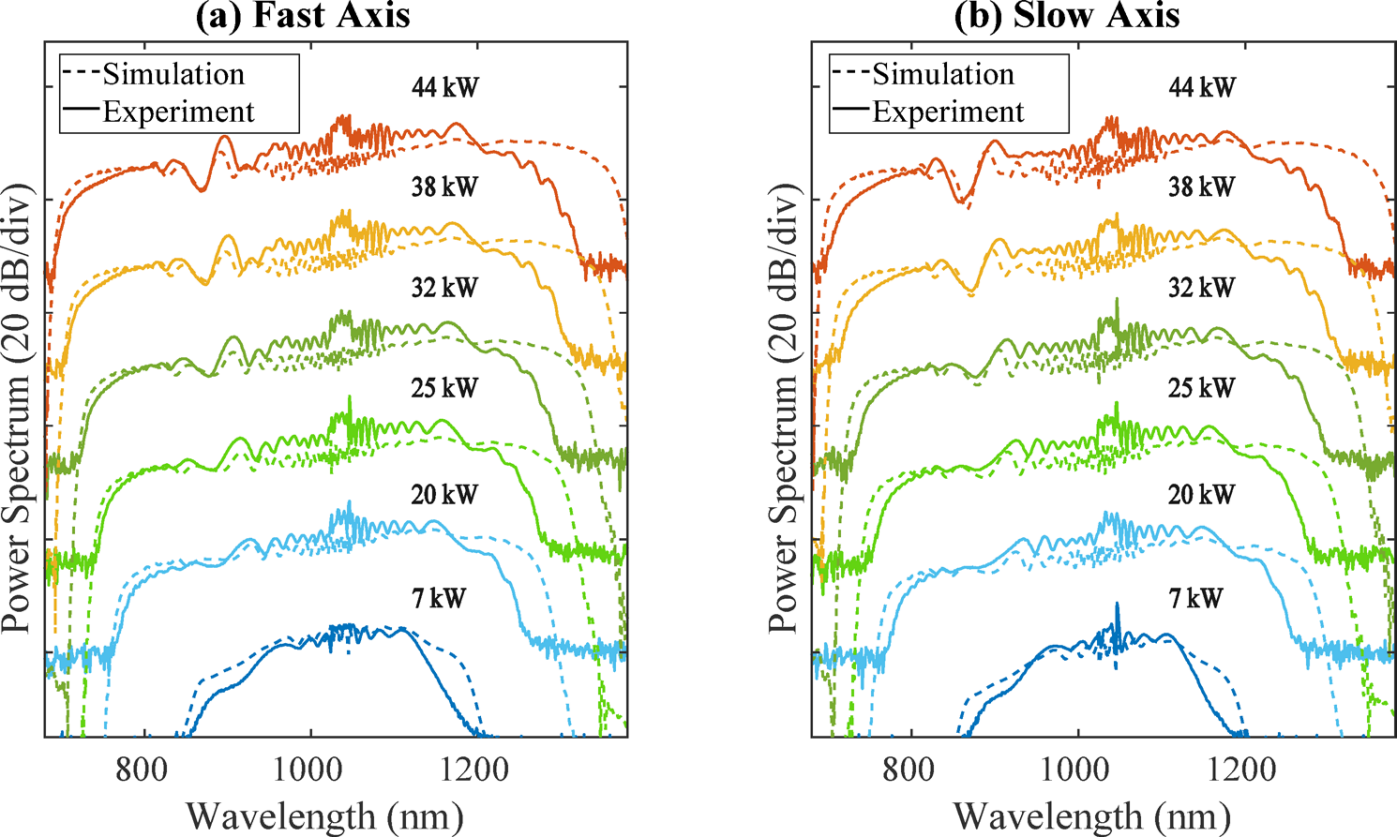
Experimental (solid) and numerical (dashed) ANDi-fibre output SC spectra (logarithmic scale, arbitrary units) along both fibre principal axes as a function of input pulse peak power, using a Ytterbium femtosecond laser pump at 1034 nm. All spectra are vertically shifted for improved clarity.

**Fig. 3:**
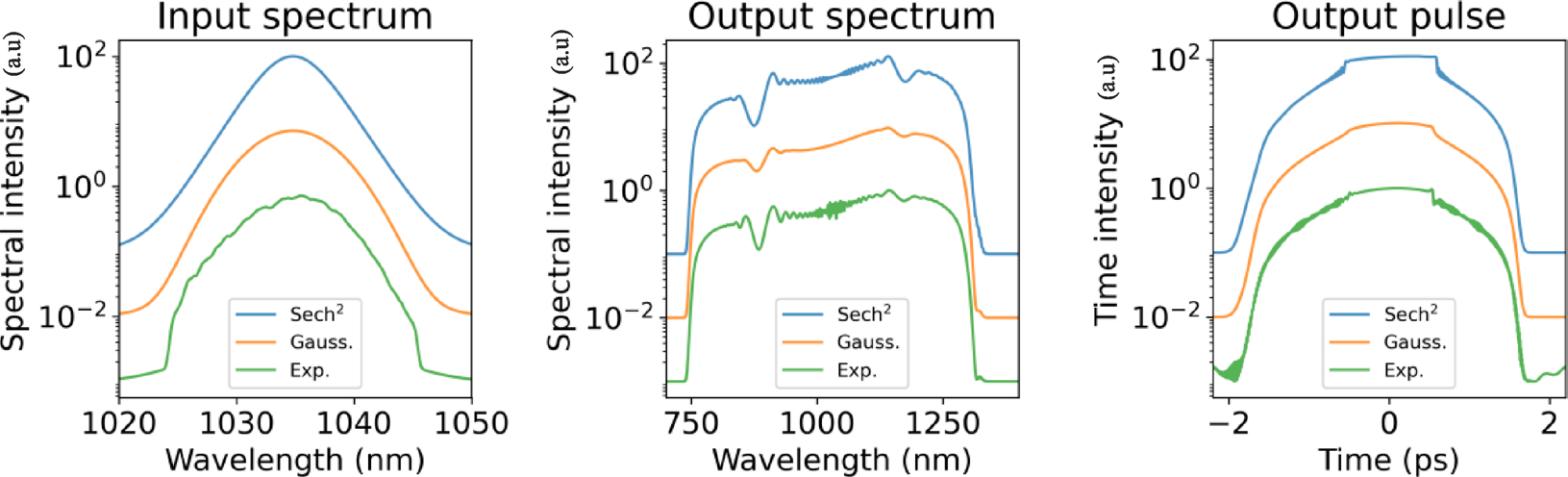
Numerical simulations showing the impact of the input pulse spectral shape on the SC output, using three input pulse spectra: a sech^2^-shaped spectrum (blue), a Gaussian-shaped spectrum (orange), and the experimentally measured spectrum (green). The y-axis uses a logarithmic scale, and the different spectral and temporal profiles are offset vertically for clarity.

We first pumped a 12.5 cm-long section of the drawn fibre using an Yb:YAG CPA femtosecond laser (Pharos PH1-SP-1mJ), delivering 175 fs (FWHM) pulses centered at 1034 nm. The resulting output was analysed using an optical scanning spectrometer (APE waveScan). Figure 2a, b show the measured SC spectra as a function of input peak power pumping along the two polarisation axes, comparing experimental data (solid lines) with numerical simulations (dashed lines). The experimental input spectrum (shown in Fig. 3, green) was taken into account in the simulations, assuming Fourier-transform-limited pulses (pulse duration of ~175 fs FWHM pulses at 1034 nm). Apart from a discrepancy in the infrared, which is attributed to the fibre confinement losses, good qualitative agreement is seen, especially in terms of spectral evolution, bandwidth and overall structure. At the highest input peak power (44 kW, corresponding to a pulse energy of 8 nJ), the SC spectrum extended from 720 nm to 1300 nm, yielding a spectral bandwidth of 580 nm at the − 10 dB level, demonstrating state-of-the-art performance^4^. Strong spectral modulations in the central spectral region from region 900–1100 nm were observed in the experimental spectra (see Fig. 2), and we used numerical simulations to determine its origin as associated with particular features in the input pulses. In particular, Fig. 3 shows GNLSE simulation results using three different input spectral shapes: an ideal sech^2^-shaped spectrum (blue), an ideal Gaussian-shaped spectrum (orange), and the experimentally-measured spectrum (green).

In all case, the input pulses were assumed to be Fourier-transform limited leading to ideal sech and Gaussian temporal profiles for the respective spectra, but for the experimental spectrum with the clipped edges, the corresponding temporal profile had weak pre- and post-pulses and degraded temporal contrast. When using input pulses based on the measured spectrum in the simulations, we are able to successfully reproduce the spectral modulations seen in experiments, as shown in Fig. 2.

#### Pumping at 1030 nm with 120 fs pulses

**Fig. 4:**
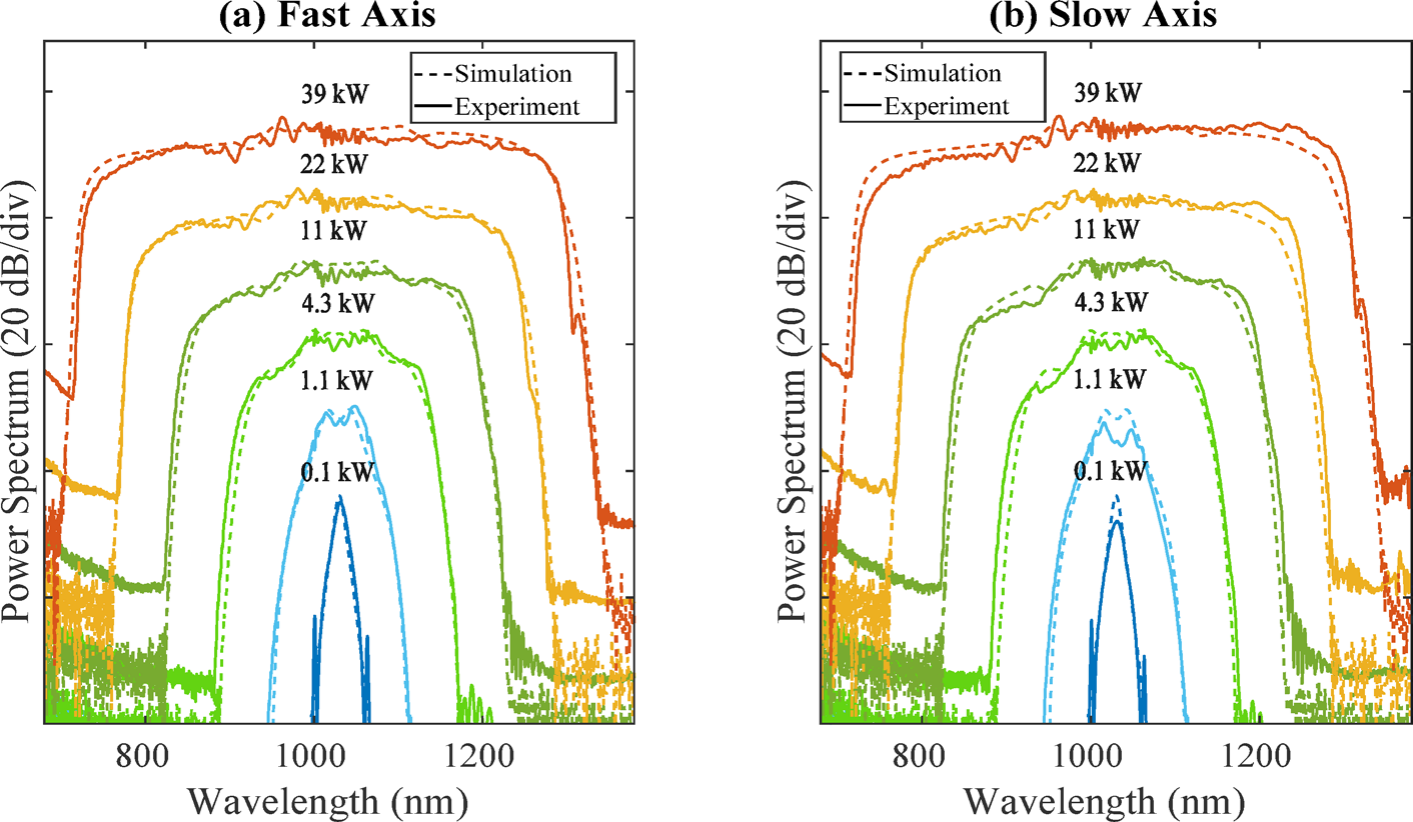
Experimental (solid) and numerical (dashed) fibre output SC spectra (logarithmic scale, arbitrary units) versus input peak power using a Ti:Sa femtosecond laser at 1030 nm when pumping the PM-ANDi fibre on (**a**) fast axis and (**b**) slow axis.

We then tested a 20-cm long functionalized PM-ANDi fibre with end-caps and fiber connectors using a tunable femtosecond Ti:Sapphire laser (Coherent Chameleon Ultra II), which delivers quasi-gaussian and chirp-free 120 fs pulses (FWHM). Figure 4 presents the experimental results obtained with a pump wavelength of 1030 nm.

As shown, the ripples across the pulse center are significantly reduced, and the supercontinuum still extends from 730 to 1300 nm. The simulated spectra shown in dashed lines using 120 fs sech^2^ pulses match very well the experimental ones. At full coupling efficiency, we measured a total SC average output power of 360 mW, corresponding to a power spectral density of 0.5 mW/nm.

#### Pump tuning from 920 nm to 1040 nm with 120 fs pulses

Next, we investigated SC generation versus pump wavelength with a tunability from 920 to 1040 nm.

**Fig. 5:**
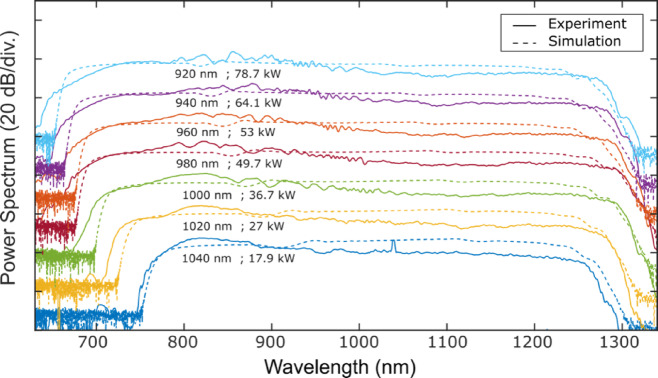
Experimental and numerical output SC spectra (logarithmic scale, arbitrary units) for different pump wavelengths, ranging from 920 to 1040 nm, and peak powers using a tunable Ti:Sapphire femtosecond laser.

Experimental results are shown in Fig. 5 together with numerical simulations. A maximum achievable bandwidth of 650 nm (from 650 to 1300 nm is achieved) when using a pump wavelength at 920 nm with 78.7 kW pulse peak power. Indeed due to the parabolic profile of the dispersion curve the SC generation is robust against changes of pump wavelength and is even broader thanks to the rising peak intensity due to the Ti:Sapphire gain profile.

#### Comparison with a commercial PM-ANDi fibre

We then compared the experimental results obtained with our designed PM-ANDi PCF to those from a commercial polarisation-maintaining ANDi fibre from NKT Photonics (Model NG-1050-NE-PM)^15^, which achieves polarisation maintenance using two borosilicate rods. The NKT fibre features a relative hole size of d/Λ = 0.45, a small hole-to-hole pitch of 1.44 µm, and a nonlinear coefficient of 26.8 W^−1^ km^−1^ at 1040 nm. Its MDW is centered at 1040 nm. The fibre cross section can be seen in Ref.^13^.

**Fig. 6:**
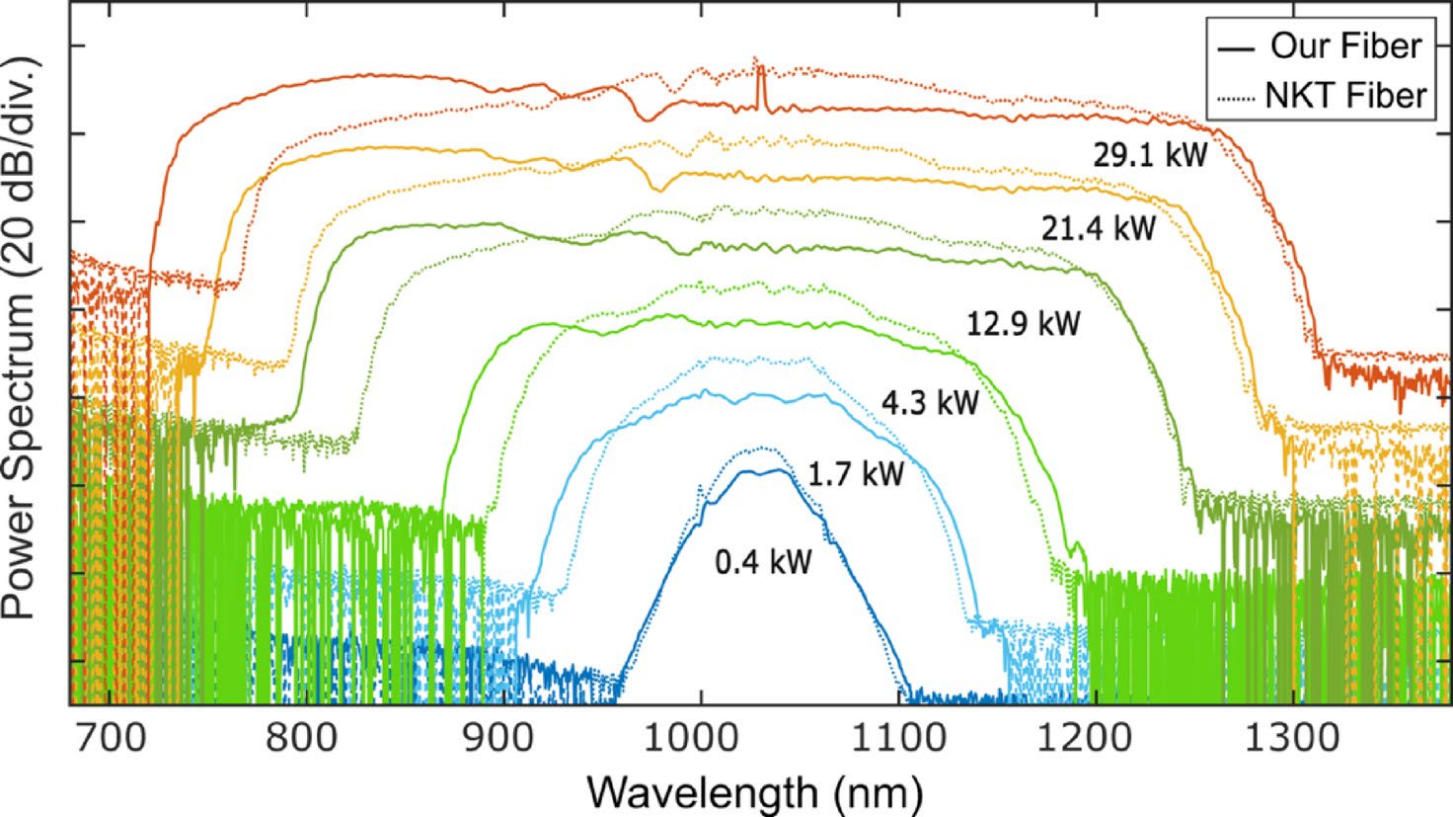
Comparison of the experimentally measured SC output spectra (log scale, arbitrary units) generated in the new PM-ANDi fiber (solid lines) and a commercial PM-ANDi fiber (dashed lines) as a function of input peak power using a Ti:Sa femtosecond laser.

Figure 6 shows a direct comparison of the SC spectra generated in both fibers under identical conditions: the same peak power and a fiber length of 25 cm. While both fibers support broadband SC generation, the new PM-ANDi fiber exhibits a slightly broader spectrum, particularly on the short-wavelength side. This improvement is attributed to its smaller effective modal area (4.37 µm^2^ vs. 4.96 µm^2^), its lower group-velocity dispersion at the pump wavelength (9.16 × 10⁻^3^ ps^2^ m⁻^1^ vs. 1.12 × 10⁻^2^ ps^2^ m⁻^1^ at 1030 nm), and its standard outer diameter, which facilitates alignment and efficient coupling into the fiber.

### Noise measurements

The stability and coherence of SC sources are critical for a wide range of applications, including frequency metrology, spectroscopy, and imaging. In particular, SC generation in ANDi fibers is well known for its ultra-low noise and high spectral coherence^1–6^, making it especially attractive for demanding use cases. In this work, we characterised the noise properties of the generated SC using three complementary approaches: (i) shot-to-shot stochastic fluctuations of the SC relative intensity noise (RIN)^19^, (ii) shot-to-shot fluctuations of the SC spectral optical phase^20–22^, and (iii) a full noise analysis performed in the microwave domain^23,24^.

#### Relative intensity noise (RIN) measurements

To record the shot-to-shot fluctuations of the SC spectral intensity, we employed the dispersive Fourier transform (DFT) technique^19^. This technique enables real-time, pulse-to-pulse SC spectral measurements in the time domain by temporally stretching the pulse using a normal dispersion fibre such that its stretched temporal profile mimics its spectrum. In our experiment, we utilized a 232-m-long dispersion-shifted fibre (DSF) with a net normal dispersion of − 24 ps/nm, to stretch the input pulses, enabling real-time observation of the SC spectrum on an oscilloscope. At the DSF output end, the temporally stretched spectrum was measured through fast detection systems, ensuring adequate temporal resolution for the time–frequency mapping associated with the DFT measurement technique. Here, we used a 5-GHz InGaAs biased photodiode (Thorlabs, DET08CFC) connected to a 12-GHz real-time oscilloscope (Agilent DSA 91204A). The DSF fibre dispersion and slope are β_2_ = 36.5 ps^2^/km and β_3_ = 4.9 ps^3^/km, respectively.

**Fig. 7:**
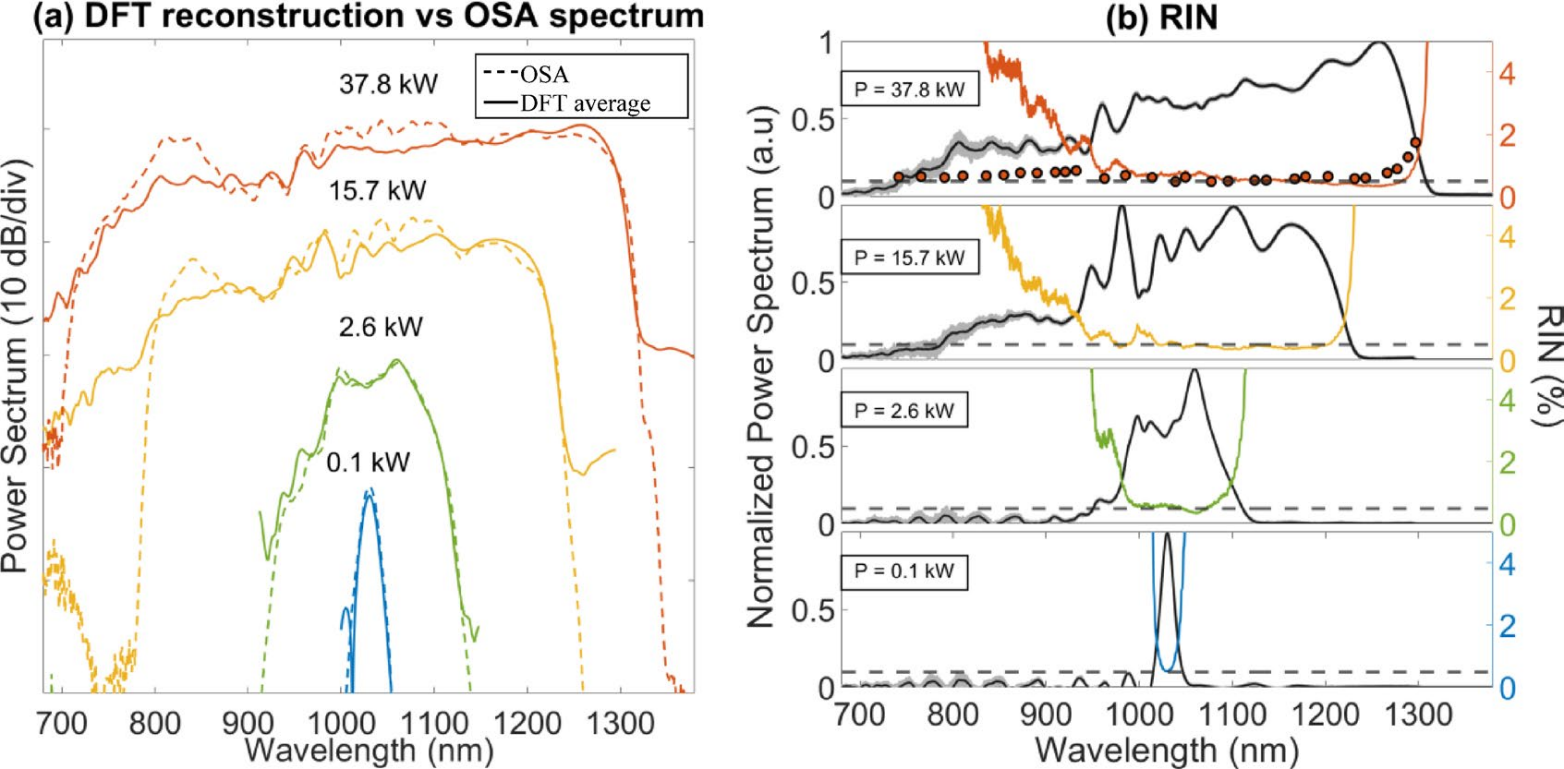
(**a**) Experimental DFT spectra (solid lines) compared with OSA spectra (dashed lines) for increasing input peak power when pumping the fibre along the fast axis. (**b**) Corresponding RIN spectra (color lines) for the same increasing peak powers. Light gray lines are the shot-to-shot measured statistical fluctuations of the SC and black curves correspond to the average DFT spectra. The horizontal dotted-line marks 0.5% which is the noise floor of our system corresponding to the combination of our initial pump laser source RIN and the detection system limit.

This gives an equivalent spectral resolution close to 8 nm. The DFT results are shown in Fig. 7a for 292 pulses, where the corresponding average DFT spectrum (solid lines) is compared with OSA spectrum (dashed lines). As can be seen, the agreement is very good, validating the DFT method. From the DFT spectra, the RIN was then calculated according to the following equation : $RIN\left( \omega \right) = \sqrt {\langle\left( {I\left( \omega \right) -\langle I\left( \omega \right)} \right\rangle)^{2} \rangle} / \langle I \left( \omega \right)\rangle$, where $I\left( \omega \right)$ is the spectral intensity. RIN spectra for increasing input peak powers are shown in color in Fig. 7b. We measure RIN values as low as 0.5% across the SC bandwidth (see dashed horizontal lines), except in the short-wavelength region. This deviation is mainly attributed to the stretching fibre and the reduced photodiode sensitivity in that range. To verify this, additional RIN measurements were performed without the stretching fibre, using a tunable grating filter scanned across the SC spectrum and a statistical analysis of the pulse-to-pulse peak power standard deviation, following the method described in Ref.^13^. The red dots in the upper part of Fig. 7b represent these measurements, obtained by filtering the SC output with a tunable 25 nm bandpass grating filter and analysing 292 photodiode impulse responses. An excellent agreement is observed in the long-wavelength region between the two measurement techniques. Overall, our results show a nearly constant RIN of about 0.5% across the full SC bandwidth, confirming the absence of noise amplification during the SC generation process.

#### Optical phase noise measurements

**Fig. 8:**
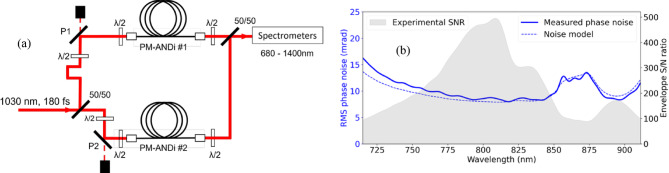
Optical phase noise measurements. (**a**) Experimental setup. P1 and P2: thin-film polarisers. (**b**) relative phase noise of the two SCs as a function of wavelength (blue solid line), phase noise model (dashed blue line), signal-to-noise (SNR) ratio of the retrieved fringe envelope (gray area), defined as the ratio of the ensemble average to ensemble standard deviation, of 1000 successive single-shot measurements.

In the absence of a broadband phase reference, direct measurement of shot-to-shot spectral phase fluctuations was not possible. Instead, we evaluated phase reproducibility by recording the spectral interference between two SC sources generated in identical fibres under identical input pulse conditions. This method, known as Bellini-Hänsch interferometry^20^, was previously adapted to investigate the phase reproducibility of filamentation in bulk crystals^14^. The experimental setup, shown in Fig. 8a, consists of two identical PM-ANDi fibres integrated in a balanced Mach-Zhender interferometer. The single-shot spectral interference between the two SC outputs is analysed using a discrete Hilbert transform^21^, enabling extraction of the relative phase fluctuations as a function of both shot number (i.e., time) and wavelength. By analysing 1000 consecutive spectra, we quantify the statistical phase stability across the accessible wavelength range. As emphasized earlier, this technique does not yield an absolute measure of spectral phase stability but instead evaluates the stochastic phase noise inherent to the SC generation process. Our measurements shown in blue in Fig. 8b reveal phase noise with a standard deviation of 8–15 mrad over the 700–925 nm range and 7–10 mrad over the 1100–1330 nm range (not shown). These values match a noise model shown in dashed blue including the passive phase stability of the interferometer (measured routinely to ~ 6 mrad rms at 1030 nm and extrapolated further) and the effect of both shot noise and read noise on the phase retrieval algorithm. From a classical fluctuating phase model^20^, the latter is approximated by the ratio of the shot-to-shot standard deviation of the retrieved fringe envelope to the average envelope, that is 1/SNR where SNR is the signal-to-noise ratio of the retrieved fringe envelope (See gray area in Fig. 8b). From these experimental measurements and from this noise model, we conclude that the level of determinism of SC generation in PM-ANDi fibres is so high that no stochastic phase (including relative timing jitter between the two fibres) can be meaningfully measured.

#### Electrical noise measurements

To examine the noise characteristics in more detail, we measured the phase and amplitude noise of the SC source in the microwave domain using a Microchip 53100A phase-noise analyser referenced to a dual 100-MHz oscillator. The dual-reference configuration enables cross-correlation measurements with a phase-noise sensitivity below − 160 dBc/Hz for Fourier (offset) frequencies between 5 kHz and 1 MHz. Experimentally, the SC output was spectrally filtered into 25-nm bands using a tunable diffraction grating and detected with a 5-GHz InGaAs biased photodiode (Thorlabs DET08CFC). A 50-Ω load was connected to the photodiode via the DC port of a bias-tee, while the AC port was low-pass filtered to suppress harmonics above the 80-MHz carrier corresponding to the laser repetition rate. The RF power was kept constant at 1 dBm for all measurements by adjusting the optical power incident on the photodiode and using a 14-dB gain RF amplifier, ensuring accurate and repeatable noise characterisation. The amplified signal was then fed into the noise analyser and compared with the dual-reference oscillator noise floor. Single-sideband (SSB) phase- and amplitude-noise power spectral densities (PSDs) of the 80-MHz beat note were recorded from 1 Hz to 1 MHz.

**Fig. 9:**
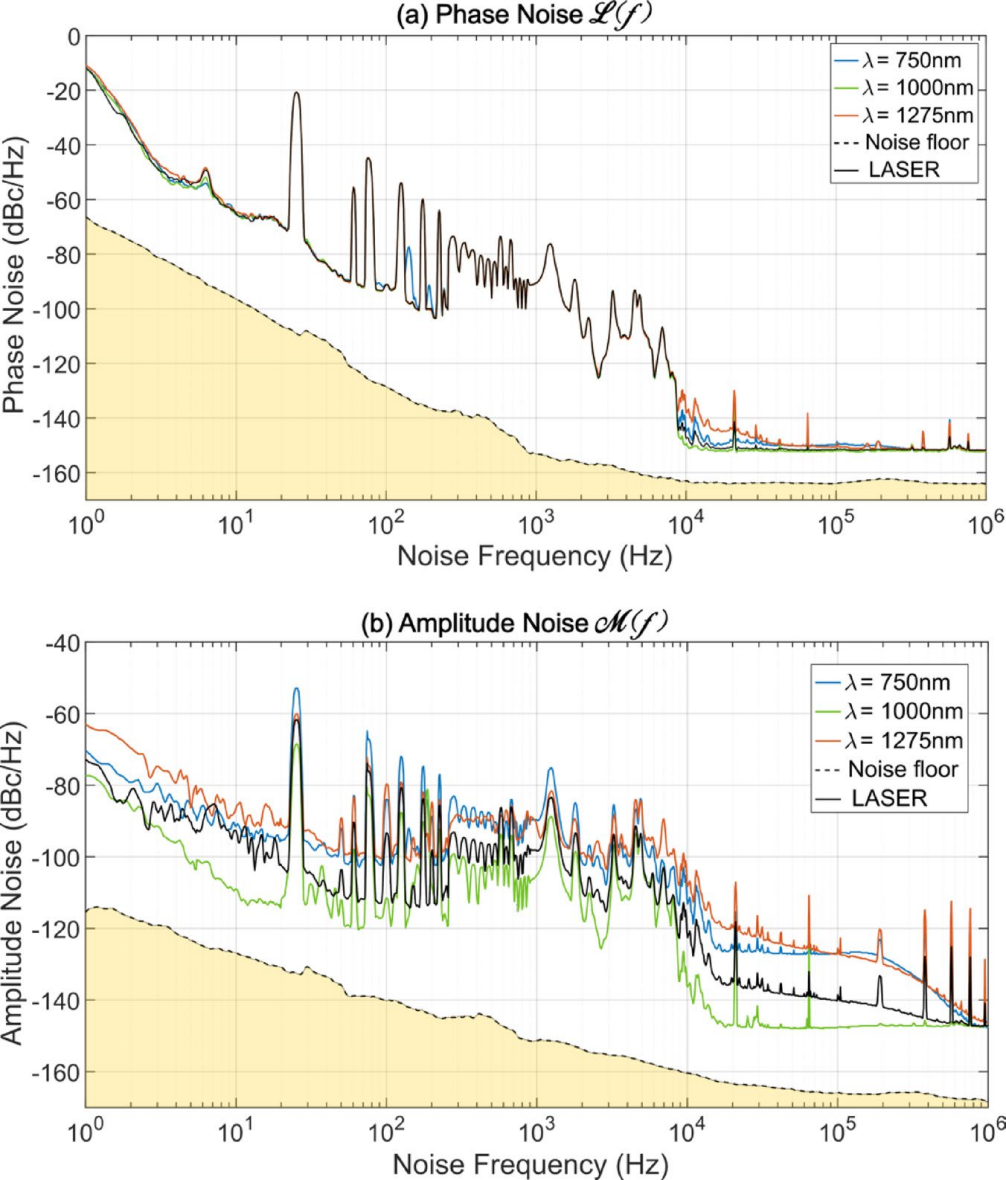
(**a**) Phase and (**b**) amplitude noise power spectral density (PSD) of the 80-MHz beat signal measured over the 1 Hz–1 MHz range using a Microchip 53100A phase-noise analyser. The yellow region indicates the noise floor set by the two reference 100-MHz oscillators. The solid black curve corresponds to the input femtosecond pump laser, while the coloured curves show the noise spectra of the 20-nm spectrally-filtered ANDi-fiber supercontinuum output at three different center wavelengths, as shown in the inset.

The measured phase- and amplitude-noise PSDs are shown in Fig. 9a, b, respectively. We compare the input laser noise (solid black curves) with the SC noise at the spectral centre and edges (coloured curves) as well as with the analyser floor (dashed black curves). As expected, the free-running mode-locked Ti:Sa femtosecond laser exhibits the characteristic low-noise profile of frequency-comb sources, featuring several peaks due to acoustic and thermal technical-noise processes and a phase-noise level of approximately − 150 dBc/Hz at a 10-kHz offset, corresponding to the RF amplifier’s white-noise floor at this power level^22^. Similar features are observed across all spectrally filtered SC bands. Notably, the SC output maintains phase-noise levels around − 150 dBc/Hz beyond 10 kHz, whereas the amplitude noise increases significantly toward the spectral edges. This is fully consistent with established ANDi-SC theory, which predicts higher noise near the edges and reduced noise near the centre of the spectrum^11,18^.

## Discussion

The slight discrepancy between the target pump wavelength (1030 nm) and the minimum dispersion wavelength (MDW), located near 1010 nm in Fig. 1a, originates from inherent fabrication tolerances. In microstructured fibers, small variations in air-hole diameter or pitch during the drawing process can induce measurable shifts in the dispersion profile, making nanometer-level control of the MDW particularly challenging. Nevertheless, the impact of this ~ 20 nm offset on supercontinuum (SC) performance remains limited. As previously shown in Ref.^4^, when the dispersion profile is relatively flat around its minimum, the exact positioning of the MDW is not critically restrictive. The fiber therefore remains fully suitable for broadband SC generation even when the pump wavelength does not coincide precisely with the MDW. Indeed, as illustrated in Fig. 5 and supported by Ref.^1^, octave-spanning SC generation can still be achieved for significantly larger pump–MDW mismatches, provided the overall dispersion landscape remains favorable. The main consequence of such a mismatch is a slight spectral asymmetry: the broadening tends to extend more strongly toward the MDW side, while remaining efficient in the opposite direction. In our case, despite the MDW being located near 1010 nm, pumping at 1030 nm produces a flat and broadband spectrum, confirming the robustness of the design.

We have also quantified the polarization-maintaining performance of the fiber by measuring the polarization extinction ratio (PER) across the generated SC bandwidth. The new fiber exhibits a PER of approximately 15 dB over the full spectral range. For comparison, under similar experimental conditions, the commercial NKT fiber shows a slightly higher PER of approximately 17 dB across the same bandwidth^13^. Although the PER of the new fiber is marginally lower, it remains fully compatible with stable PM operation over the entire SC spectrum.

Finally, regarding operational parameters, it is important to emphasize that in the all-normal dispersion regime the achievable SC bandwidth is governed primarily by the input peak power rather than by the pulse duration itself. As demonstrated in previous studies^1^, similar bandwidths can be obtained for different pulse durations provided the peak power is maintained, with longer pulses simply requiring longer propagation lengths to fully develop the nonlinear broadening. In our experiments, using 120 fs and 180 fs pulses, the full spectral bandwidth is reached within the first ~ 10 cm of fiber. Extending the fiber length beyond this point does not significantly increase the bandwidth, as the spectrum is already fully developed and undergoes only minor reshaping. Although increasing the peak power can in principle further broaden the spectrum, we observe a sub-linear, nearly logarithmic dependence of bandwidth on peak power in our operating regime, indicating that the supercontinuum is close to saturation. Consequently, further power increase provides only marginal spectral extension while introducing greater sensitivity to coupling fluctuations and injection instabilities, which can degrade stability and reproducibility. For these reasons, the selected fiber lengths (≤ 25 cm) and peak powers (≤ 80 kW) represent a practical compromise between bandwidth, stability, and operational robustness.

## Conclusion

In summary, we have developed a novel polarisation-maintaining all-normal-dispersion (PM-ANDi) photonic crystal fibre featuring a simplified structural design based on two enlarged central holes. This configuration eliminates the need for conventional stress rods, enabling a compact and practical 120 µm outer diameter. The fibre exhibits a near-parabolic dispersion profile centered around 1030 nm and achieves state-of-the-art supercontinuum generation with broad, flat spectra spanning from 630 to 1350 nm. Its compatibility with various femtosecond laser sources and outstanding noise performance, validated through pulse-resolved DFT-based relative intensity noise and phase noise measurements, demonstrate its strong potential for integration into high-performance ultrafast laser systems. The proposed fibre design is particularly well-suited for advanced applications such as dual-comb spectroscopy, coherent frequency comb combining, nonlinear imaging, and f-to-2f interferometry.

## Data Availability

The data that support the findings of this study are available from the corresponding author upon reasonable request.
